# Environmental Regulation in Evolution and Governance Strategies

**DOI:** 10.3390/ijerph20064906

**Published:** 2023-03-10

**Authors:** Guangyuan Xing, Youheng Zhang, Ju’e Guo

**Affiliations:** 1 School of Economics and Finance, Xi’an Jiaotong University, Xi’an 710061, China; 2 School of Management, Xi’an Jiaotong University, Xi’an 710049, China

**Keywords:** environmental regulation, environmental policy, development trend, climate change, governance strategies

## Abstract

Environmental regulation faces theoretical and practical research challenges on global scale, due to differences such as language and policy environment. Research reflects the beneficial exploration of scholars, policymakers, and enterprises in the cognitive and behavioural norms of economic development, environmental protection, and social governance. This study demonstrated that the relevant research is motivated by the enaction of environmental regulations and discussed the influence of relevant research on the evolution of environmental regulations. Under the assumption that environmental regulations are consistent with related research, this study used 9185 papers in the field of environmental regulation from 2000 to 2019 to construct a research network panorama and explored the development and revelation of environmental regulation. The results revealed that environmental regulation research is motivated by the introduction of policies, and regulation is influenced by research evolution with the path of ‘competitiveness-technological change-innovation.’ In addition, after the twenty-first session of the Conference of the Parties (COP21), the number of studies increased significantly, with the USA in the leading position in the research field. Furthermore, governance strategies were inspired by real events, including the increasing concern with climate change, regional research preferences, and the promotion of information disclosure. These results suggest that environmental governors should focus on climate change, localisation, and mechanisms of information disclosure.

## 1. Introduction

Environmental regulations are a series of policies and laws formulated to limit pollution [1]. In general, environmental regulations are classified into three categories: command-and-control (also called direct regulation), market-based (also called economic instruments), and voluntary (also called soft instruments). Command-and-control regulations enforce control of environmental behaviour through standards and prohibitions [2]. Laws are a familiar manifestation. Market-based regulation refers to economic measures used to regulate related activities, such as taxation. Voluntary regulation includes commitment and agreement.

With the increasing prominence of environmental issues, environmental management focuses on various targets, including air pollution, water pollution, and biodiversity. With stronger recognition of environmental issues, the complexity of environmental management has increased, particularly with climate change at the centre of worldwide environmental governance. Researchers face the theoretical challenges of classical environmental management theories, such as Porter’ hypothesis, the environmental Kuznets curve, and the pollution haven hypothesis, in new global environmental governance. Policymakers face tangible challenges from complex environmental issues, such as carbon neutrality.

Representing the tangible evidence of environmental management, environmental regulation has been a favourable policy tool in practice. In investigating the development of environmental regulation from a global perspective and identifying patterns and inspirations based on it, several problems block inference, such as language, political environment, and legal system. Therefore, researchers focus on specific regions in most instances. To avoid these errors due to the particularity of policy tools, we chose academic literature as the observation object. English-based academic language breaks through the language barrier of understanding policy to a certain extent, and the large amount of data and complex structure are sufficient to support detailed analyses.

Environmental regulations have a pivotal role in multiple disciplines. For instance, they are strongly associated with performance and competitiveness in the economic and management field [3,4]. In environmental science, regulation is a tool to address environmental damage [5]. In the engineering field, environmental regulations are supported by data, viability, and so on [6]. Moreover, environmental regulations are a key concern within the fields of biology and medicine and usually regarded as impacting the development of organisms [7]. 

Existing studies have focused on environmental regulation, including the development and mechanism of environmental regulation itself. O’Riordan [8] analysed the changing characteristics of environmentalism, determined how it may affect a wide range of public policies, and predicted the future direction of environmental regulation. Alló and Loureiro [9] summarised existing assessments of climate change mitigation and adaptation policy preferences using a global meta-analysis. Ribeiro and Kruglianskas [10] proposed an effective principle to control the quality of environmental regulation using a literature review. Furthermore, some focused on a specific research object or tool, including the measurement and implications for business-oriented environmental regulation [11], modelling methods for environmental policy [12], the application of Stakeholder Analysis (SA) [13], and examining the industry and environmental policy through previous research on the impact of environmental policy for manufacturing firms [14]. Moreover, some studies investigated the relationship between environmental regulations and other issues. Iraldo et al. [2] reviewed the literature on the relationship between environmental regulations and competitiveness and summarised methods for defining and measuring the impact of environmental regulations on competition and market power. Borsatto and Bazani [15] evaluated the correlation between environmental regulations and green innovation and found it generally positive. Aragòn-Correa et al. [16] focused on how mandatory and voluntary environmental regulations affect environmental strategies and performance. Shao et al. [17] concluded that the relationship between environmental regulations impacted corporate innovation due to corporate competitiveness and sustainable development of regional economies. However, studies on the general concept of environmental regulations are lacking. 

Based on the complex disciplinary context of studies on environmental regulations, a scientometric method using a large amount of data is a workable scheme; this approach could help break the limitation of a certain issue or discipline vision. With the development of network and information science, the scientometric method is increasingly adopted in various articles, using a knowledge map to describe the status of the research field clearly and comprehensively. Martinez et al. [18] reviewed studies on environmental footprint, materialising the increasing interest in the domain. Olawumi and Chan [19] studied sustainability and sustainable development utilising the scientometric method. He et al. [20] proposed the topic and trend evaluation of agricultural waste management using this method. Qiu et al. [21] adopted the bibliometric method to investigate green aviation industry. 

The present study used scientific methodology to verify the consistency between environmental regulations and research in this field, showing that their evolutions are consistent in characteristics and time. This study aimed to structurally identify the current research situation and development status, determine researchers with prominent contributions and significant recognition, describe the situation for research cooperation, summarise the research development pattern, and grasp the future development trends. This study sought to understand the status, characteristics, and trends of environmental regulations on the global scale.

Compared with previous research, the novelties of this study are its focus on research methodology and scale. On the methodology, we chose the research as a medium to study environmental regulation. After a demonstration about the consistency between two, we study the status, trends, and characteristics of environmental regulation using scientometric analysis and visualization tools. On the topic of scale, the study evaluated previous research on environmental regulation in the macroscale and described environmental regulations in a broad sense. It could be meaningful for overcoming the barriers, including language, culture, and so on, when studying environmental regulation worldwide.

This study addressed the following key goals: (1) verify the conjecture of the consistency between environmental regulation research and environmental regulation; (2) observe the development and focus of environmental regulation worldwide; and (3) enlightenment of environmental governance.

## 2. Materials and Methods

We used the Web of Science (WOS) database in this study. This library is one of the most authoritative academic websites, particularly the Web of Science Core Collection. The criteria were: (1) themes = ‘environmental policy’ or ‘environmental regulation’ or ‘pollution policy’ or ‘pollution regulation’; (2) database = Science Citation Index Expanded (SCI-EXPANDED), Social Sciences Citation Index (SSCI); (3) time span = ‘2000–2019’; (4) document types = ‘article’ or ‘review’. Literature type was limited to journal articles in English. 9259 search results were retrieved, including 8689 articles and 570 reviews. The accurate number of records was reduced to 9185 after removing 29 articles or reviews published online in 2019 but not published officially, and 45 articles or reviews published online in 2019 then published officially in 2020.

We used two software tools for scientometric analysis and for visualising the results: CiteSpace and VOSviewer.

CiteSpace is a software system for information visualisation, developed by Dr. Chaomei Chen [22] in the School of Information Science and Technology at Drexel University, that can measure and analyze scientific literature data. The method offers an effective way of revealing the knowledge graph and intuitively displaying the panoramic view of scientific information. Specifically, it is useful for identifying key studies, research hotspots, and frontier directions in a certain field. We used CiteSpace to conduct a variety of analyses of academic publication data on environmental regulations, including subject, co-citation, and keywords analyses.

VOSviewer is another scientific tool for knowledge mapping developed by Centre for Science and Technology Studies (CWTS) and funded by Leiden University in The Netherlands [23]. It was used for subject, collaboration network, and keyword analyses in this study.

## 3. Consistency between Environmental Regulations and Relevant Researches

To overcome the barriers in researching environmental regulations across nations, we tried to utilize the academic literature information as the medium. For this to happen, the consistency between environmental regulations and relevant research must be first proved. Therefore, we will try to verify the mutual influence between environmental regulation and the research focused on it.

### 3.1. Research Is Inspired by Regulation Enaction

**Figure 1:**
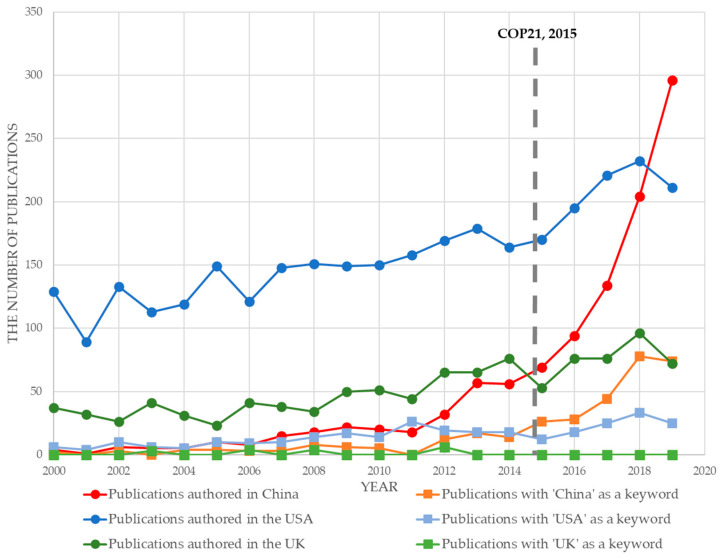
Temporal distribution of publications, authored by nations, and with national names as keywords.

The twenty-first session of the Conference of the Parties (COP21) inspired researchers dedicated to environmental regulation worldwide; this can be seen from the total number of publications following the event. In addition to global environmental events such as COP21, regional regulation enaction would have the same impact on related researchers. The explosion in the number of publications authored by nations and including national names in keyword data were related to environmental regulation development. The number of studies indicated that new environmental regulation enaction attracted the attention of scholars, particularly native scholars. Figure 1 shows the rapid growth of publications authored in China and temporal distribution.

Before 2014, the number of annual publications authored in China was never over 60, and the number of annual publications with ‘China’ as a keyword was under 40. These figures did not grow with time. However, by 2019, the former number increased to nearly 300 and maintained a significant growth rate, and the latter grew remarkably. While the annual numbers in the USA and UK stabilised at certain ranges, Chinese researchers focused on specific factors. We inferred that the key factor was the new Environmental Protection Law approved in April 2014, which is frequently mentioned in background sections of articles with ‘China’ as a keyword after 2014.

Since the 1980s, environmental laws in China gradually improved, including the Environmental Protection Law of the People’s Republic of China (PRC) (for trial implementation) in 1979, the most authoritative environmental regulation of China at the national level, the Water Pollution Control Act of the PRC in 1984, the Air Pollution Prevention and Control Law of the PRC in 1987, and the Environmental Noise Pollution Prevention Law of the PRC in 1996. Through other policy initiatives, Two Control Zones (TCZ), provincial policies, and industry environmental standards were enacted, and the environmental regulation system in China was established. The Chinese government established the third version of Environmental Protection Law in 2014 (EPL’14) through four reviews over two sessions of National People’s Congress (NPC), twice as often per period when compared to a typical law revision [24]. EPL’14 is considered the strictest environmental protection law in history due to its important additions, including increasing the severity of consequences for violating environmental protection laws, expanding the scope of environmental impact assessment, and allowing nongovernmental organisations to sue polluters. It increased the accountability of polluters and government bodies/officials, increased public disclosure, supported public interest lawsuits, and contained protection for whistle-blowers [24,25,26]. In addition to playing a role as the research background, several studies examined EPL’14 directly, focusing on environmental regulation and corporate financial performance and considering EPL’14 an empirical research object or natural experiment [27].

The results suggest that researchers are inspired by the enaction of regulation. First, new regulation or its review is a new empirical object that could provide evidence for theories. In addition, it is a telling sign that the degree of emphasis on the environment in the nation changed; this motivates researchers, especially native researchers, to investigate the practical issues of environmental regulations. Furthermore, enaction of regulation is a natural experiment, where researchers can contrast the data before and after and analyze the impact of the environmental regulation to testify concepts. Moreover, current regulations may contain new structures or governance initiatives, that were unexamined by existing theories; therefore, researchers are inspired to take theoretical innovation and push theories forward.

### 3.2. Research Is Inspired by Relevant Research

We adopted a scientometric tool to identify hot keywords in recent years and proposed possible future directions for research topics in the domain.

**Figure 2:**
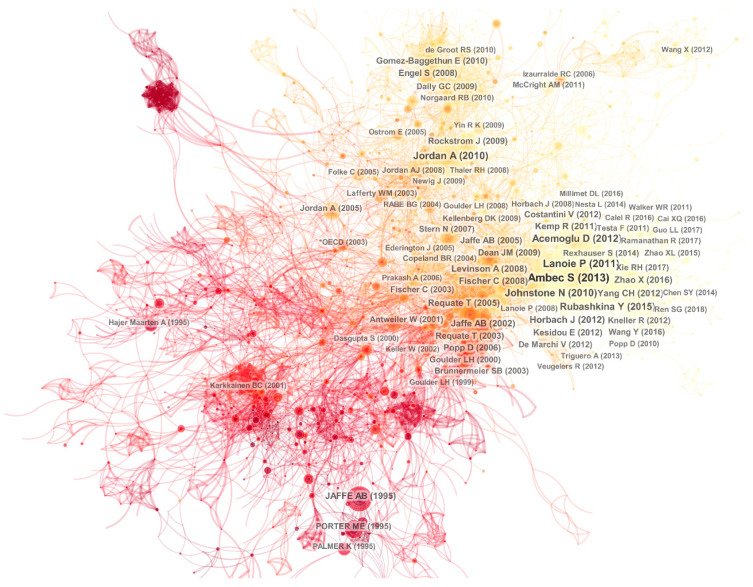
Co-citation network of publications on environmental regulations from 2000 to 2019.

When two or more authors and their literature were cited by subsequent articles at the same time, these authors and studies were considered to constitute a co-citation relationship. Using CiteSpace, we built a co-citation network to indicate how research on environmental regulation developed during the previous 20 years. Figure 2 provides the co-cited network of publications on environmental regulation. The overall theme gradually shifts with time along the path ‘competition-technology change-innovation’. The colours of nodes and connections in the network are related to their first citation and first co-citation. Darker colours indicate earlier times.

There is a critical node in the red structure in the lower-left part of the network. This is the classic article published by Jaffe et al. [28], where the author discussed the relationship between environmental regulations and competitiveness. In addition, Porter and Linde [29] produced a new conception of the environment-competitiveness relationship, known as the Porter Hypothesis, that developed the view that reducing pollution emissions could increase productivity and the innovation triggered by environmental regulation could reduce cost [30]. Palmer et al. [31] evaluated environmental standards and cost in the same year, the critical time point when researchers shifted from considering environmental regulation as absolute cost. The majority of the co-cited literature was on the topic of competitiveness. Therefore, we defined the period as the competitiveness stage.

Another representative article authored by Jaffe et al. [32], discussing the relationship between environmental policy and technological change, led researchers to examine technological change. The group with this article as the central node was the most tightly connected section of the co-cited network.

In 2010, Johnstone et al. [33] investigated how environmental policies affect technological innovation. Lanoie et al. [34] explored the relationship between environmental regulation and innovation to support the ‘weak’ version of the Porter Hypothesis. Research focus on environmental regulation evolved into innovation, and a series of studies on the Porter Hypothesis were produced, including by Ambec et al. [35], who reviewed the pivotal theoretical basis and empirical evidence of the Porter Hypothesis. Studies on the Porter Hypothesis developed. Research on environmental regulations and innovation continued to be valued by researchers, as shown in the yellow structure in the right part of the co-cited network.

Environmental policy integration (EPI) was another branch of research. Jordan and Lenschow [36] reviewed studies on EPI in the two decades before 2010, which attracted some attention; however, studies on EPI have dwindled in recent years.

**Figure 3:**
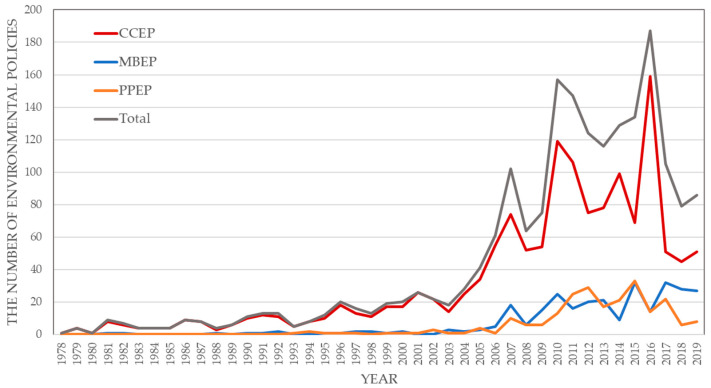
Environmental policy number in China during 1978 to 2019 [37].

The evolution of environmental regulation types in theory is confirmed in practice. The trend towards monetisation and commodification of ecosystem services is a transformation from classical economics of the original concept to natural benefits acting as use values. Guoxing Zhang et al. (2022) collected all environmental policies issued by the Chinese government from 1978 to 2019. Each policy was separated into three types: command-control environmental policy (CCEP), market-based environmental policy (MBEP), and public participation environmental policy (PPEP), as Figure 3 shows. 

We can observe obvious changes in the types of environmental regulation policies in China from 1978 to 2019. First, most were of the CCEP type. After 2006, MBEP and PPEP gradually increased. By 2019, these two types of soft environmental regulations nearly halved. With rapid economic growth, China’s 40-year policy change is the epitome of the evolution of environmental regulation. Policymakers’ expectations for soft environmental regulation have strengthened, and this trend is still growing.

## 4. Development Status of Environmental Regulations Reflected by the Researches

Based on the consistency between environmental regulations and relevant research, we could find the development status of worldwide environmental regulation by studying the distribution, contribution, collaboration, and academic genres of research on environmental regulation.

### 4.1. Distribution of Literature

To measure the contributions to environmental regulation between 2000 and 2019, we observed published data from articles or reviews. We presented the temporal and the international distribution of research quantity. We assessed contributors to the research based on authors of publications and the authors’ institutes and nations. Environmental regulations are a broad and common academic concept, studied across multiple disciplines. Therefore, we adopted the WOS Categories tag in publications data to reveal the disciplinary coverage and distribution. Journal, institution, and author information are all meaningful parts of publication data, and we utilized them to identify the most influential contributors.

**Figure 4:**
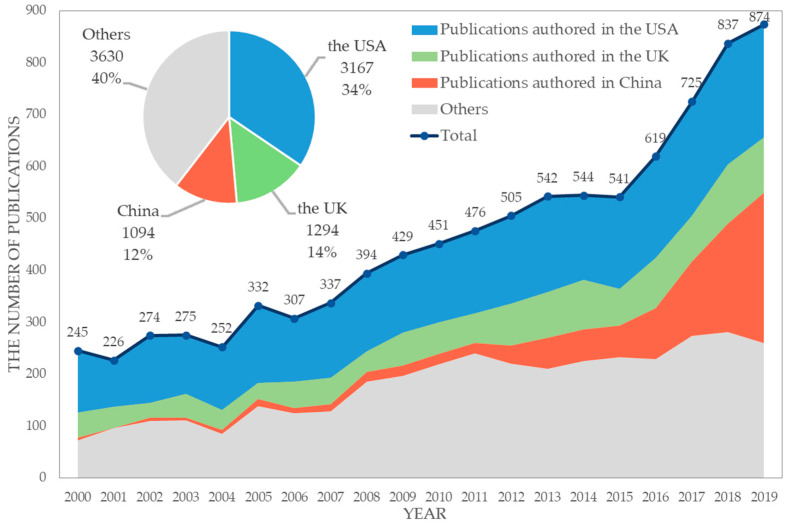
Temporal and international distribution of academic publications on environment regulation from 2000 to 2019.

The number of academic publications on environmental regulation steadily increased across our period, as shown in Figure 4. We observed that the number of publications has exploded since 2015, the year when COP21, also known as the 2015 Paris Climate Conference, was held. COP21 has historic significance; it was the first time a legally binding universal agreement on climate had been achieved, with the aim towards keeping global warming below 2 °C. Since the agreement, acting on climate change was ‘nationally determined’ in parties, leading to revolutionary consequences [38]; this was reflected in essential timing of research on environmental regulations.

A total of 127 nations participated in the 9185 studies on environmental regulation published from 2000 to 2019. The ten nations with the greatest number of articles and reviews published are presented in Table 1. These publications together accounted for 97.10% of the total research contribution, and the top three nations together occupied 60.48%, indicating that research on environmental regulation is concentrated in a few nations. The gap between first and second was over 20%; this shows that the contribution by the USA was dominant in the field.

**Figure 5:**
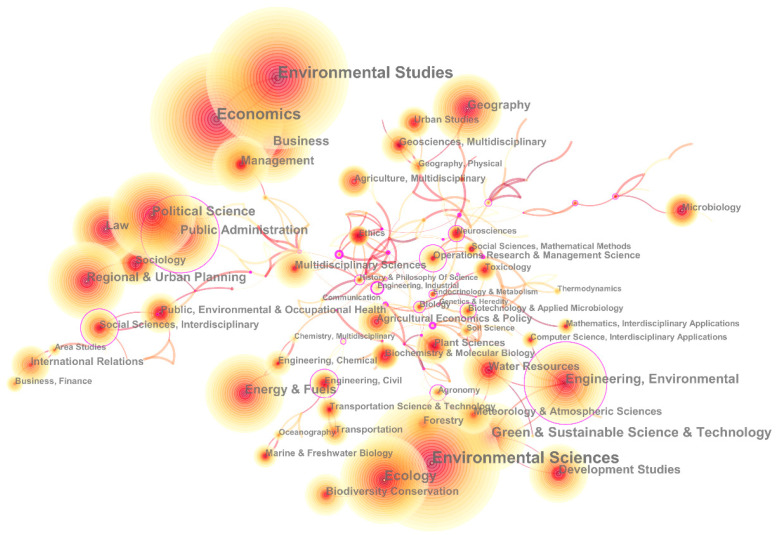
Disciplinary network of academic publications on environmental regulation from 2000 to 2019.

Studies on environmental regulations are covered by multiple disciplines; hence, we observed disciplinary distribution in the way a discipline co-occurred network was built using CiteSpace based on Web of Science Categories (WC) tags in the publication data, as shown in Figure 5. In the network, nodes represent the WC tags, and links mean interdisciplinary research. The size of each node depends on the number of publications with the WC tag, and the strength of each link depends on the count of publications with both two tags. The colour of nodes and links in the network correspond to the time when they were first generated between 2000 and 2019. Deeper colours indicate earlier times. Specifically, the outermost layer of a few nodes could be purple, showing the high betweenness centrality of the node; this is regarded as the pivotal node or hub node in general.

The most contributing discipline was ‘Environmental Sciences’, involving 2771 publications. The following were ‘Environmental Studies’ with 2754 publications and ‘Economics’ with 2335 publications. Other disciplines containing more than 500 publications included ‘Green and Sustainable Science and Technology’, ‘Ecology’, ‘Engineering, Environmental’, and ‘Political Science’. Viewing the network, we identified several co-occurrence disciplinary groups. The first group was composed of ‘Environmental Studies,’ ‘Economics’, ‘Business’, and ‘Management’, where researchers studied environmental regulation as a factor in economic, business, and management activities [39,40,41]. The second group consisted of ‘Environmental Sciences’, ‘Engineering, Environmental’, ‘Green and Sustainable Science and Technology’, ‘Ecology’, ‘Development Studies’, ‘Water Resources’, and ‘Plant Sciences’, where environmental regulations were considered as a controlling activity in biology and ecology process [42] or policy subject in environmental issue [43]. In the third group, composed of ‘Political Science’, ‘Public Administration’, ‘Law’, ‘Sociology’, ‘Regional and Urban Planning’, ‘Public, Environmental and Occupational Health’, and ‘International Relations’, environmental regulations were the major research object as a type of law or policy on environment field [44], and several cross-discipline works were found. ‘Regulation’ is a specialised word in biology, referring to the influences on and control of gene expression. This was the primary reason we could see disciplines related to biology in the network. Moreover, there were the group including ‘Energy & Fuels’, ‘Engineering, Chemical’, and ‘Engineering, Civil’, the group including ‘Transportation Science and Technology’, and ‘Transportation’, and many other groups.

### 4.2. High-Yield Contributors

The high-yield journals were similar to the disciplinary categories of publications on environmental regulation; these journals primarily focused on Environmental Sciences, Ecology, Business, and Economics. A total of 11 journals published over 100 articles and reviews on environmental regulation in two decades. Table 2 illustrates the ten journals with the most contributions in the field. Ecological Economics contributed the most, focusing on expanding and integrating the interface between ecosystems and economy.

Using to the institute information of the publications, that shows the institutes the authors work for, we obtained a list of the high-yield institutes across two decades, as shown in Table 3. Institutes belonging to the USA occupied half of the Top 10 list; this was consistent with the fact that the USA had a dominant position in the research field. The most contributing institute was the University of California, Berkeley. Furthermore, other campuses of the University of California made considerable contributions to the research field of environmental regulation, including Davis, Los Angeles, and Santa Barbara. If we regard the campuses of the University of California as one institute, its academic advantage was more prominent. This reinforced that the USA not only had an advantage in the total number of publications in the research field but also several high-yield research institutes. Although China had a relatively large number of publications, only one institute, the Chinese Academy of Sciences, appeared in the top 10 list. Therefore, China’s research advantages were unconcentrated, and the dominant number of publications was due to the advantage in the numbers of institutes and researchers. Similarly, Germany ranked fourth in the number of publications; however, no specific research institute entered the list.

By counting the publication produced by each author, we obtained a list of the ten most productive authors, shown in Table 4. Four were from the USA, but not from the most contributing institute, the University of California, Berkeley. We could speculate on the current research status on environmental regulation in the University of California, Berkeley, arguing that there were numbers of researchers engaged in this field with a few of works but a lack of researcher leaders. Except for the University of Maryland, all institutes in Table 3 exhibited the same situation, indicating that more academic experts work for institutions that are not in dominant positions. One of Johannes Urpelainen’s articles, that discussed the relationship between path dependence and political competition, was published in the American Journal of Political Science in 2013 and has been cited 70 times in the Web of Science Core Collection [45].

### 4.3. Collaboration

Researchers tend to choose their localities as study objects, as previously mentioned. With this accepted, we sought to understand how researchers from different localities choose their co-operators. To identify the status of research collaboration for environmental regulation research among nations, institutes, and authors, we evaluated the published articles as collaborations between them. We built collaboration networks on different node levels (international, institutional, and authorial) based on the corresponding tags in the literature data using VOSviewer.

**Figure 6:**
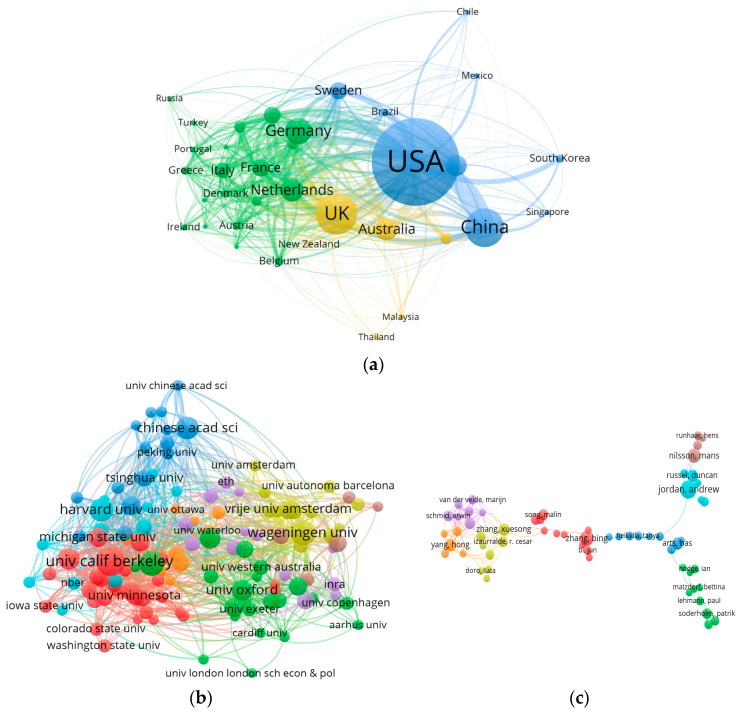
Collaboration network in research on environmental regulation from 2000 to 2019. (**a**) International collaboration network; (**b**) Institutional collaboration network; (**c**) Authorial collaboration network.

There were 37 nations with more than 30 publications. Figure 6a presents the international collaboration network. We grouped the nodes in the network into three clusters based on a clustering algorithm, showing the situation of international collaboration. The first international group with close collaboration was composed of 20 nations, including Germany, The Netherlands, France, and Italy, and was plotted using green. What stood out in cluster #1 was a broad and balanced collaboration relationship among these nations, and most of them were European nations. Unlike the first collaboration group, the other two consisted of fewer countries with more simple and clear collaboration relationships. The USA and China head cluster #2 in blue. In this group, the USA and China, who were the first and third biggest national contributors, respectively, had intimate collaboration relationships. Therefore, it was the most remarkable collaboration relationship in the research field of environmental regulation. The collaboration network structure of cluster #3 in yellow was sparser, only composed of eight nations, where the primary collaboration relationship was between the UK and Australia. In addition to the key nodes in each collaboration group, we also needed to focus on joint nodes between them; these play pivotal roles in interconnection. Sweden was the joint point of cluster #1 and #2, which means researchers in Sweden collaborated with their inherent partner and conducted studies with researchers in cluster #2. Joint points, such as Sweden and New Zealand, promoted collaboration between different international groups.

The institutional collaboration network reflects the cooperation between research institutions in research on environmental regulation between 2000 and 2019. A total of 88 institutes with more than 30 publications were clustered into six groups, as shown in Figure 6b. Cluster #1 in red is represented by the University of California, Berkeley, and primarily includes US research institutes, showing a series of important cooperative relations among American research institutes. The University of Cambridge, University of Oxford, University of Gothenburg, and University of Helsinki formed cluster #2 in green. It principally comprised research institutes of European nations. The University of British Columbia belonged to cluster #2 and cooperated closely with cluster #1, based on institutes of the USA, and acted as an important connection point between the two collaboration groups. The primary nodes of cluster #3, in dark blue, reflected the close cooperation relationship between Chinese scientific research institutes and Harvard University. 

There were 250 authors with more than five academic publications on environmental regulation, of which 60 were connected. The collaboration network was visualized in Figure 6c and clustered into eight groups. According to the network, the collaboration between researchers was not close in the field, and only a few authors occurred in the collaboration network. Among them, Chinese and American scholars had the closest collaborative relationships.

### 4.4. Academic Genres

**Figure 7:**
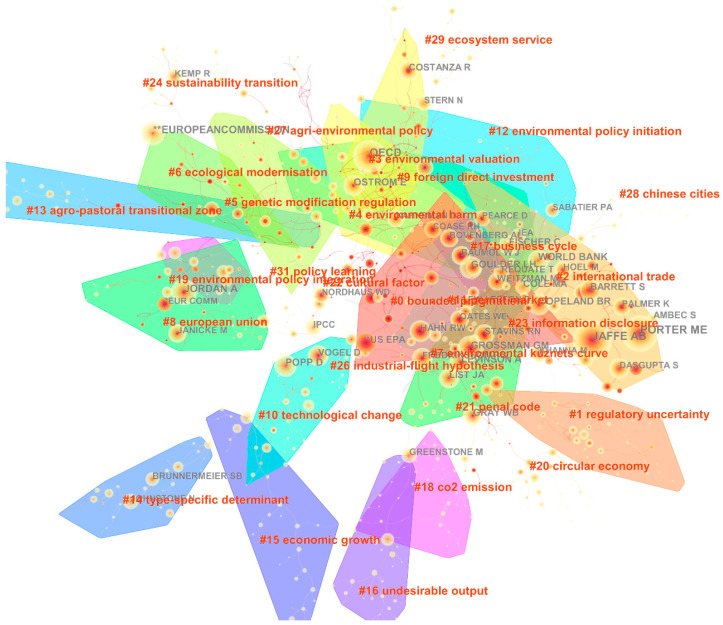
Co-citation network of authors in research on environmental regulation from 2000 to 2019.

To identify commonly recognised authors in the research field, we established a co-citation network of authors, as can be seen in Figure 7. Using it, we revealed the relationship between the most recognised authors and their co-citation situation in the research field. Then, academic genres holding related viewpoints and focusing on similar research topics were obtained. We used the clustering algorithm and the label extracting algorithm, the log-likelihood ratio (LLR), proposed by CiteSpace, to cluster and extract labels from titles, abstracts, and keywords. There were 32 clustering results obtained, that represented the similarity of author group research to a certain extent. Appendix A presented the details. 

We indicated academic leaders according to research topic. Jaffe AB, Porter ME, and Ambec S were referenced centrally in cluster #2 in relation to research topics about international trade, cross-section tests, and the sustainable energy sector. In cluster #8, which contained research topics about the European Union, new environmental policy instruments, and eastern Europe, Jordan A, Janicke M, and the European Commission were the most recognised authors. When we observed the Organisation for Economic Co-operation and Development (OECD), we found that it played a vital role in four clusters (#3, #4, #9, and #12), and was popular among several related academic genres. In particular, a few authoritative scholars, led by the Intergovernmental Panel on Climate Change (IPCC), could not be clustered. This may be because the results of their research are cited in a wide range of areas, which was somewhat approved.

## 5. Environmental Governance Strategies from the Reality

Environmental regulations have a strong political attribute and regional label; therefore, research on them inherits these characteristics and would be reflected in the studies from recent years. We focused on the uniqueness of research on environmental regulation, exploring which events were related to research efficiency. We proposed environmental governance strategies from these findings.

### 5.1. Topic Detection

Using keyword data, we identified the distribution of themes to discern the future of the research topic. The list of the top occurring keywords is summarised in Table 5.

**Figure 8:**
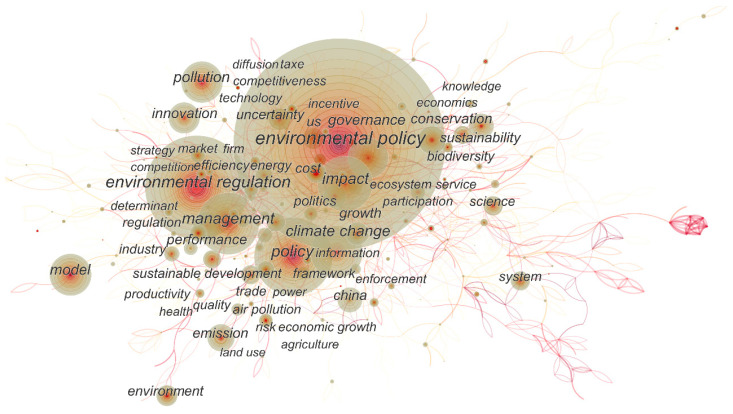
Co-occurring network of keywords in research on environmental regulation from 2000 to 2019.

We illustrated a keyword co-occurring network, shown in Figure 8. This revealed the relationship and distribution of the research topics. Studies on ‘environmental policy’ were surrounded by ‘governance’, ‘politics’, ‘climate change’, and ‘ecosystem service’, which were in the position of national and social governance. Research on ‘environmental regulation’ was another core node, with related research on ‘management’, ‘performance’, ‘market’, ‘efficiency’, and ‘competition’, which were from the perspective of company management. Research on ‘sustainability’, ‘conservation’, and ‘biodiversity’, was sufficiently relevant, and belonged to environmental science and biology. Based on the rings of nodes in the network, we identified that the later ones, such as ‘innovation’ and ‘climate change’, were emerging academic hotspots on a certain scale.

To identify the developing trends of research on environmental regulation, we measured the burst strength of keywords using CiteSpace and obtained several meaningful results. We identified a key time period that ran from 2013 to 2015, as illustrated in Appendix B. A total of 14 keywords had a burst time distribution that lasted until 2019, which suggests that they were the most attractive topic and that studies on them continue to emerge rapidly, including ‘carbon dioxide emission’, ‘restoration’, ‘climate change adaptation’, ‘eco-innovation’, ‘environmental Kuznets curve’, ‘directional distance function’, and ‘legitimacy’. The focus on ‘climate change’ beginning in 2015 was related to COP21, and it is likely to be a persistent hotspot in the future. Kuznets curve is the curve of income distribution changes with the economic development process, proposed by Kuznets [46]. The environmental Kuznets curve proposed by Grossman and Krueger [47] usually refers to the similar relationship between environment and the economy. Since 2015, the popularity of the environmental Kuznets curve as an empirical research object or research methodology has exploded. This indicates that, with the accumulation of evidence, the environmental Kuznets curve attracted more attention and was accepted by more and more scholars. Another meaningful word was ‘panel data’, which is one of the most recognised data types applicable to the field.

The bursting national and regional names were related to the environmental regulation development; this attracted the attention of scholars, particularly native ones. We observed the appearance of a few national and regional names in the list, including ‘UK’, ‘Germany’, ‘Africa’, ‘Europeanisation’, and ‘California’, which revealed that the empirical objects change by time due to certain facts. We examine two dominant nations contributing to research on environmental regulation, the USA and China, in the following sections.

### 5.2. Focus on Climate Change

**Figure 9:**
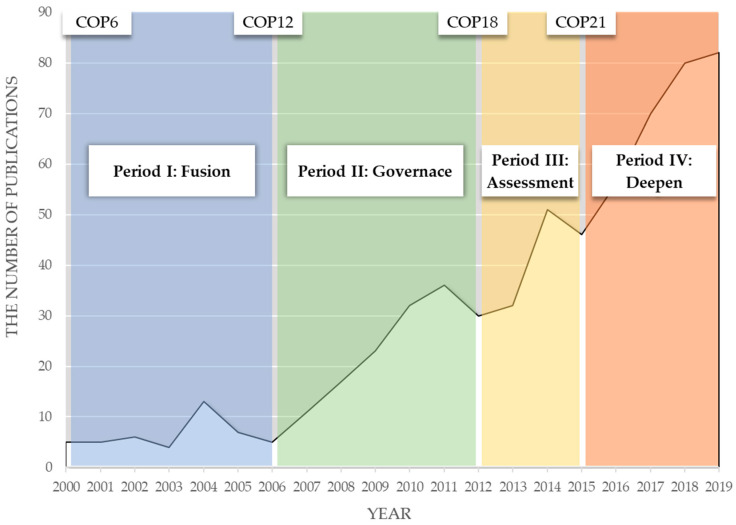
Temporal change in publications on climate change and environmental regulation.

**Figure 10:**
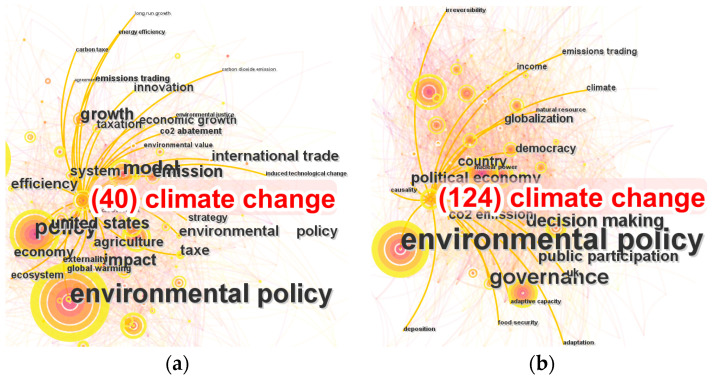

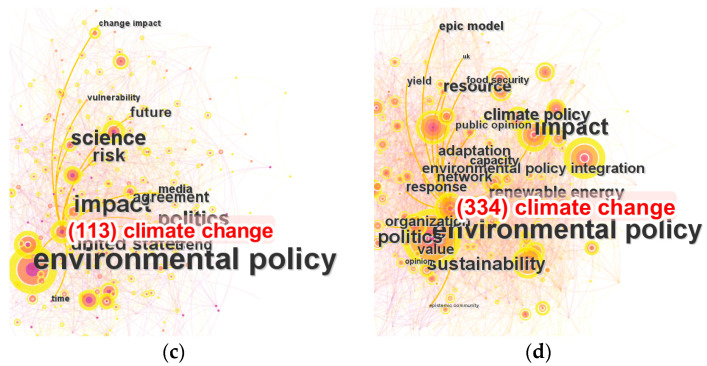
Topic network of related literature in each period. (**a**) Period I: Fusion, 2000–2005; (**b**) Period II: Governance, 2006–2011; (**c**) Period III: Assessment, 2012–2014; (**d**) Period IV: Deepen, 2015–2019.

Climate change was not always the focus of research on environmental regulation, but gradually became a topic with rapidly increasing attention. A total 611 items of our sample included ‘climate change’ in the title, abstract, or keywords. The number change of literature related to climate change by year is illustrated in Figure 9. As the high-profile theme in the field of environmental regulation, the number of publications on climate change increased to 85 in 2020, 109 in 2021, and 85 in 2022. We divided the research on environmental regulation about climate change into four periods. To further examine the characteristics of research in different periods, we computed the topic network of climate change literature in each period, as shown in Figure 10.

Global climate governance in the 21st century started from the sixth session of the Conference of the Parties (COP6) as a conference without consensus, which indicated the divergence in climate realities and the challenge to environmental regulation theories. In Period I, 2000–2005, only 40 studies included environmental regulation and climate change. Researchers began to focus on both climate change and environmental regulation in this period, and the topics were variant. Period I could be viewed as the fusion stage of climate change and environmental regulation research. Some research directions were formed, such as empirical research on carbon tax [48] and the criticism of climate politics [49]. As there were few truly implemented environmental regulation on climate change at that time, the relevant research objects were limited. The most cited studies focused on energy-economy-environment models. At the method level, researchers began to improve climate change models with policy and innovation variables [50,51] and tried to apply integrated assessment frameworks [52].

With the steady growth of the research on environmental regulation, the quantity of cross research tripled in the second six-year period. The Nairobi work program was proposed in the twelfth session of the Conference of the Parties (COP12) to help developing countries improve their ability to cope with climate change. The effect of its governance is shown by the 124 studies published in Period II, 2006–2011. More environmental regulations were employed, especially in developed countries, which provided the objects for research on governance issues responding to climate change. Researchers tried to summarize the few governance experiences of developed countries. The governance for climate change in countries was different in case studies. The top-down targeted adaptation approach in the UK generated anticipatory action, which marked transition to a well-adapting society [53]. The climate mitigation actions in the USA were bottom-up, which indicates that a decentralized environmental policy structure is effective for climate change [54]. In the period, researchers also studied other problems in governance to meet climate change, including social justice dilemmas [55] and multilevel governance [56].

The eighteenth session of the Conference of the Parties (COP18) legally ensured the second commitment period of the Kyoto Protocol, and a few countries exited, which could have inspired people to review the impact and wish of implementation. Researchers contributed 113 studies on climate change and environmental regulation in Period III. They studied the process of self-examination in response to climate change and attempted to provide reliable assessments to facilitate it. The researchers’ focused on the assessment of interaction between climate change and food [57], water [58], and energy [59] from policy perspectives. Internal relationships that acted to address climate change also gained attention, including public concern [60,61], political polarization [62], science–policy interaction [63], and environmental assessment [64].

COP21 launched a prosperous era of research on climate change and environmental regulation. There were 334 related studies in Period IV. Based on the research on basic problems published during the first three periods, Period IV contains more in-depth research on climate change and environmental regulation. The critical issues included how to enact socio-technical transition pathways in environmental regulation frameworks [65], how to use the energy system model to support policymaking [66], and how to drive public support for innovation based on policies [67]. After the ‘Fusion-Governance-Assessment-Deepen’ pathway of cross research, climate change was a significant proposition for environmental regulation. With the practice of environmental regulation addressing climate change in more countries, especially in developing countries, policymakers need more research to support the addition of climate goals to environmental regulation systems. We foresee that there will be more research on climate change using the paradigm of environmental regulation.

### 5.3. Preference of Local Research

As environmental regulations are enacted by governments in each nation or region, it is vital for researchers to consider political background, especially when discussing empirical cases. The gap between native researchers and others includes data gaining, language ability, understanding of regulation, and research interest, which leads the actuality that the emphasis on research theme differs between nations. Thus, an obvious fact could be noted that local researchers are more active on the topic that concern their own nation.

**Figure 11:**
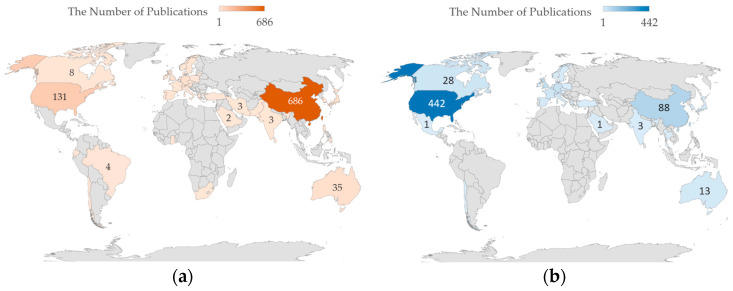
International distribution of publications with national names as keywords. (**a**) national distribution of publications with ‘China’ as keyword; (**b**) national distribution of publications with ‘US’ as keyword.

We counted the international distribution of authors in the publications on the research with clear national labels to verify local research preference. For multiple authors of one publication and multiple institutes for one author, the non-repetitive national information of each author was recorded once. For example, there is an article with three authors as follows: Author 1 (Institute in Nation A, Institute in Nation A), Author 2 (Institute in Nation A, Institute in Nation B), Author 3 (Institute in Nation A); in this case, Nation A will be counted twice, and Nation B will be counted once. The statistical results about publications with ‘China’ and ‘US’ as keywords are plotted in Figure 11.

In all research publication data on environmental regulation between 2000 to 2019, 332 publications focused on China; the article keywords contain the word ‘China’. Through the statistical approach, the result was 1112 counts of 40 nations, which could be observed on the map. The maximum distribution point was China, which was more than half. If there was no difference between native researchers and others, the international distribution of publication with ‘China’ as keyword followed the overall distribution; in actuality, US researchers accounted for 34.48% and Chinese researchers accounted for 11.91%, which did not reflect the truth. The actual situation was that native researchers were the most productive contributors, which means local research was one of the important characteristics of research on environmental regulation. In the result of publications with ‘US’ as a keyword, the USA was the most productive contributor to its local research. Moreover, foreign scholars’ interest differs in China and the USA; we found that Canadian researchers are more likely to research environmental regulation in the USA than in China. The reason might be related to the degree of similarity between Canada and the USA when compared to Canada and China, and the degree of close in academic exchanges between two sets.

Environmental governors should know that international actions or experiences need to be deeply localized in combination with actual conditions and the policy environment. Local researchers are usually more reliable because they enter into the research field with the local background. Moreover, policymakers are always supposed to keep an eye on the relationship between environmental regulation and the local economy, society and culture.

### 5.4. Promotion by Information Disclosure

Adequate information disclosure is vital support to researchers. Information disclosure reform by a government or the establishment of an information system can often promote research on environmental regulations. We took the fine particulate matter (PM_2.5_) data of China as an example to illustrate the relation between information disclosure and research promotion on environmental regulation.

**Figure 12:**
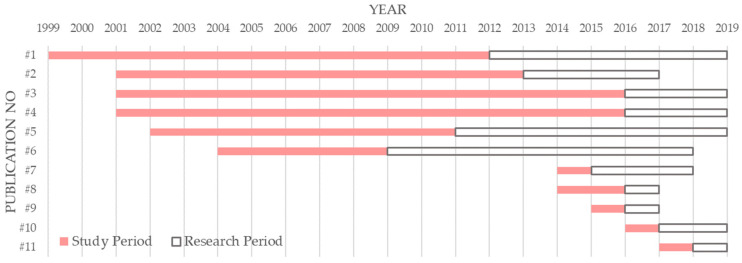
Study period and research period statistics of publications with ‘PM_25_’ or ‘PM_2.5_’ as keywords focusing on China.

In 2011, a national PM_2.5_ monitoring program was implemented in China. This program planned for four steps of implementation: monitoring PM_2.5_ for 31 capital cites of provinces before 2012, another 113 major cities before 2013, all the county level cites before 2015, and a new air quality standard being executed in 2016 [68] (the Air Quality Index, or AQI). Therefore, China started PM_2.5_ monitoring and disclosure of the data in 2012. This new data did not belong to the Air Pollution Index (API), which was replaced by AQI; as such, this is a suitable observation subject for how information disclosure impacts research focus. We filtered the publications that study Chinese data based on ‘PM_2.5_’ or ‘PM_25_’ as keywords in our dataset and obtained 11 articles, whose titles and data sources are listed in Table 6. We also measured the ‘study period’ and ‘research period’ of each filtered publication, as seen in Figure 12. The former is the time of the data used in research, and the latter is the time from the end date of the data to the publication date.

The data sources of publication #1 [72], #2 [73], and #6 [74] were both from the research results published by van Donkelaar et al. [69] at the Socioeconomic Data and Application Center at Columbia University, which used data from multiple satellite instruments to infer global PM_2.5_ concentrations between 1998 and 2012. Publication #3 [75] used the Environmental Performance Index (2016) proposed by the International Geosciences Information Centre of Columbia University. Publication #4 [76] used a high-resolution satellite-based PM_2.5_ dataset proposed by C. Q. Lin et al. [70], which could be browsed at the website (http://envf.ust.hk/dataview/aod2pm/current, accessed on 26 December 2020). Publication #5 [77] utilized the research results of van Donkelaar et al. [71] as the source, also based on the satellite data. The national urban air quality real-time publishing platform of China (http://106.37.208.233:20035/, accessed on 26 December 2020, cited by #7 and #9, http://113.108.142.147:20035/emcpublish/, accessed on 26 December 2020, cited by #8) was the common source of PM_2.5_ data used in publication #7 [78], #8 [79], and #9 [80]. This data came from an online platform published by China National Environmental Monitoring Centre. To date, the platform contains AQI, PM_2.5_, inhalable particles (PM_10_), sulfur dioxide (SO_2_), nitrogen dioxide (NO_2_), Ozone (O_3_), and Cobalt (CO) data of 31 provincial-level administrative regions and 330 prefecture-level cities. The Report on the State of the Environment in China of each province (http://www.mee.gov.cn/hjzl/zghjzkgb/gshjzkgb/, accessed on 26 December 2020,) was cited by Publication #10 [81], which gradually added PM_2.5_ data since 2012. Publication #11 [82] chose more detailed data from five environmental monitoring stations in Hangzhou due to its research scale. In summary, the PM_2.5_ data sources of publication #7 to #11 are all based on direct monitoring data, and the others are based on indirect data from satellite and other instruments.

Study and research periods of two categories of publication presented in Figure 12 show that the study periods of publications #1 to #6 spanned from 2012 or before, which means direct monitoring data did not meet the requirements of researchers, as they were generated and available since 2012. When researchers only required the data after the time when China completed the PM_2.5_ monitoring system, they consistently chose the direct monitoring data for its higher credibility, as seen in publications #7 to #11. Moreover, we infer that reliable and detailed direct data motivates related research through the observation that research periods of publication #7 to #11 were significantly shorter than publication #1 to #6. Through the example of PM_2.5_ data in China, we verified that information disclosure is expected by researchers on environmental regulation, and that it could promote research.

Policymakers should understand the importance of information disclosure to environmental regulation. Construction of information measurement systems and the mechanisms of disclosure are helpful to ensure the orderly development of environmental regulation.

## 6. Conclusions

To deal with the problem of uniformly measuring and observing the development of environmental regulation on a global scale, we utilized related research literature as the representation of environmental regulation after demonstrating the consistency between the two. We adopted a scientific measurement method to evaluate environmental regulation in a broad sense. By analyzing the retrieved publication data related to environmental regulation, the global status of the research and its development trends from 2000 to 2019 were obtained. We determined the vision of environmental regulation from related research including distribution, collaboration, and academic genres. We determined the importance of related research to environmental regulation.

First, we tried to verify the consistency between environmental regulation and related research bilaterally. Through the example of EPL’14 inspiring research on environmental regulation in China, we could regard the research as a kind of academic projection. Based on the comparison between theoretical evaluation path ‘competitiveness-technological change-innovation’ and real data changes of different types of environmental regulation, we surmised the research on environmental regulation guided its development to a certain extent. Therefore, we investigated environmental regulations via related research as an object.

Then, we discussed the development of research on environmental regulation, including the distribution of the publication by time, discipline, nation, journal, institute, and author. Environmental regulation covered a wide range of disciplines, primarily including the groups tagged with ‘Environmental Studies’, ‘Economics’, ‘Business’, and ‘Management’. As for international distribution, the USA, in the dominant position, occupied 34.48% of the number of publications, followed by the UK and China ranked second and third, respectively. We believed the contributors of research on environmental regulation were concentrated in a few leading nations. The distribution of high-yield institutes and authors also corroborated that the USA occupied a significant position, asked by the University of California, Berkeley. We obtained the international, institutional, and authorial collaboration networks. In the international collaboration network, the core was the USA. For the institutional collaboration, we obtained a network with the centre as the University of California, Berkeley, Wageningen University, the Chinese Academy of Sciences, and Oxford University. The authorial collaboration network was sparse, essentially composed of Chinese and American scholar groups.

Ultimately, we focused on what revelation we could obtain from research on environmental regulation and what would be beneficial in attempting to understand the current situation. To help better grasp current and future research hotspots, we described the distribution of research topics by the co-occurring network of keywords and detected the potential popular themes in the future by the burst strength of keywords. We first studied the research development of the topic ‘climate change’ and identified four periods: ‘Fusion-Governance-Assessment-Deepen’. Climate change is an important proposition in environmental regulation today and the need for related theory will keep growth. Then, taking China and the USA as examples, we filtered the publications with ‘China’ and ‘US’ as keywords. We counted the number of publications authored by these two nations. We believe local research is a popular pattern. We determined that information disclosure could promote research with reference to the case of the PM_2.5_ monitoring program in China promoting related research. For researchers, our suggestions are as follows. Choosing your own nation as the research object is more common and easier. Grasping the release of vital environmental regulations will help you keep up with research hotspots. Moreover, finding excellent data sources will improve your work efficiency. For environmental governors, we suggest encouraging native scholars and disclosing more available data. These tactics might increase the possibility of being the research object on environmental regulation, which is valuable for local governance.

As it was limited to our evaluation scale, this study lacks exhaustive discussions in each subdividing direction on environmental regulation. This study only adopted data from the database Web of Science Core Collection. Therefore, more research utilising scientometric methods is needed for more specific directions in the research field of environmental regulation. More extensive data are required in further studies.

## Figures and Tables

**Table 1:** Most contributing nations in research on environmental regulation from 2000 to 2019.

No.	Nation	Publications Count	Proportion
1	USA	3167	34.480%
2	UK	1294	14.088%
3	China	1094	11.911%
4	Germany	680	7.403%
5	The Netherlands	547	5.955%
6	Australia	529	5.759%
7	Canada	488	5.313%
8	France	380	4.137%
9	Sweden	376	4.094%
10	Spain	364	3.963%

**Table 2:** Journals with the highest contribution in research on environmental regulation from 2000 to 2019.

No.	Journal	Count	Proportion	Impact Factor (2019)	Impact Factor (5 Years)
1	Ecological Economics	295	3.212%	4.482	5.236
2	Journal of Cleaner Production	282	3.070%	7.246	7.491
3	Environmental and Resource Economics	258	2.809%	2.286	2.490
4	Journal of Environmental Economics and Management	202	2.199%	3.449	4.198
5	Energy Policy	190	2.069%	5.042	5.693
6	Environmental Science Policy	181	1.971%	4.816	5.335
7	Sustainability	169	1.840%	2.576	2.798
8	Journal of Environmental Management	144	1.568%	5.647	5.708
9	Land Use Policy	115	1.252%	3.682	4.151
10	Environmental Politics	114	1.241%	4.320	3.784

**Table 3:** Institutes with the highest contribution in research on environmental regulation from 2000 to 2019.

No.	Institute	Count	Proportion	Nation
1	University of California, Berkeley	119	1.296%	USA
2	Wageningen University	96	1.045%	The Netherlands
3	University of Maryland	94	1.023%	USA
4	Harvard University	90	0.980%	USA
5	Chinese Academy of Sciences	85	0.925%	China
6	U.S. Environmental Protection Agency	79	0.860%	USA
7	University of Oxford	77	0.838%	UK
8	Australian National University	75	0.817%	Australia
9	University of Cambridge	70	0.762%	UK
10	Vrije University Amsterdam	68	0.740%	The Netherlands

**Table 4:** Most contributing authors in research on environmental regulation from 2000 to 2019.

No.	Author	Count	Institute	Nation
1	Johannes Urpelainen	25	Johns Hopkins University	USA
2	P. G. Fredriksson	21	University of Louisville	USA
3	Andrew Jordan	20	University of East Anglia	UK
4	Michael J. Meaney	15	Agency for Science Technology and Research (ASTAR)	Singapore
5	Jeroen C. J. M. Van Den Bergh	13	Autonomous University of Barcelona	Spain
6	Marc J. Stern	12	Virginia Polytechnic Institute and State University	USA
7	Marco Frey	11	Scuola Superiore Sant’Anna	Italy
8	Jale Tosun	11	Ruprecht Karls University Heidelberg	Germany
9	David Popp	10	Institute of Molecular and Cell Biology	Singapore
10	Roberto C. Izaurralde	10	University of Maryland	USA

**Table 5:** Most occurring keywords in research on environmental regulation from 2000 to 2019.

No.	Keyword	Count	No.	Keyword	Count
1	environmental policy	2396	11	performance	378
2	environmental regulation	1212	12	conservation	369
3	policy	917	13	emission	354
4	management	742	14	sustainability	345
5	impact	672	15	China	332
6	climate change	651	16	growth	316
7	pollution	526	17	us	299
8	model	509	18	politics	294
9	governance	502	19	uncertainty	293
10	innovation	392	20	environment	290

**Table 6:** PM_2.5_ data source of publications with ‘PM_25_’ or ‘PM_2.5_’ as keywords focusing on China.

No.	Title	Data Source
#1	Determinants of haze pollution: An analysis from the perspective of spatiotemporal heterogeneity	The research results of van Donkelaar et al. [69]
#2	Identifying the spatial effects and driving factors of urban PM_2.5_ pollution in China	The research results of van Donkelaar et al. [69]
#3	Regional green development level and its spatial relationship under the constraints of haze in China	The Environmental Performance Index (2016) proposed by the International Geosciences Information Centre of Columbia University
#4	Decomposing the Long-term Variation in Population Exposure to Outdoor PM_2.5_ in the Greater Bay Area of China Using Satellite Observations	The research results of C.Q. Lin et al. [70]
#5	The non-linear effect of environmental regulation on haze pollution: Empirical evidence for 277 Chinese cities during 2002–2010	The research results of van Donkelaar et al. [71]
#6	Study on PM_2.5_ pollution and the mortality due to lung cancer in China based on geographic weighted regression model	The research results of van Donkelaar et al. [69]
#7	Spatiotemporal characterisation and mapping of PM_2.5_ concentrations in southern Jiangsu Province, China	The national urban air quality real-time publishing platform of China
#8	Impact and Suggestion of Column-to-Surface Vertical Correction Scheme on the Relationship between Satellite AOD and Ground-Level PM_2.5_ in China	The national urban air quality real-time publishing platform of China
#9	The Characteristics of Spatiotemporal Distribution of PM_2.5_ in Henan Province, China	The national urban air quality real-time publishing platform of China
#10	Spatial distribution of the public’s risk perception for air pollution: A nationwide study in China	Report on the State of the Environment in China (2016)
#11	Investigation on air pollution control strategy in Hangzhou for post-G20/pre-Asian-games period (2018–2020)	5 environmental monitoring stations in Hangzhou

## Data Availability

Not applicable.

## Appendix A

**Table A1:** Cluster information about the co-citation network of authors in research on environmental regulation from 2000 to 2019.

ClusterID	Size	Silhouette	Mean (Year)	Label (Top3 by LLR)
0	66	0.979	2004	adaptive ecosystem management, bounded pragmatism, regulatory penalty default
1	60	0.989	2012	regulatory uncertainty, managerial perception, contingency approach
2	59	0.975	2004	international trade, cross section test, sustainable energy sector
3	52	0.951	2004	environmental valuation, contingent valuation, economic critics
4	51	0.948	2004	environmental harm, barring exotic species, framing effect
5	49	0.933	2008	genetic modification regulation, conceptualizing joint knowledge production, success condition
6	48	0.957	2005	ecological modernisation, political influence, regulator-regulated relationship
7	47	0.963	2004	environmental Kuznets curve, pollution abatement, economic view
8	47	1	2008	European union, new environmental policy instrument, eastern Europe
9	46	0.968	2013	foreign direct investment, pollution haven, pollution-intensive industries
10	41	0.993	2010	technological change, socio-economic inertia, induced technological change
11	40	0.95	2006	permit market, green political landscape, Californias proposition
12	39	0.991	2010	local media, environmental policy initiation, policy punctuation
13	39	1	2009	agro-pastoral transitional zone, land-use change, epic model
14	36	0.993	2013	type-specific determinant, contingency perspective, innovation capacity
15	34	0.986	2017	economic growth, influencing factor, sustainability characteristics
16	34	1	2015	undesirable output, data envelopment analysis, environmental efficiency
17	34	0.966	2007	the business cycle, power sector, directing technical change
18	33	0.973	2017	co2 emission, economic growth, dynamic relationship
19	31	0.982	2010	environmental policy integration, earth system interaction, land use
20	30	0.967	2013	circular economy, voluntary cleanup program, environmental performance
21	30	0.945	2007	penal code, global warming battle, the judicial objective function
22	28	0.974	2003	cultural factor, physical activity, African American women
23	28	0.992	2004	gray area, information disclosure, green light
24	27	1	2011	sustainability transition, sustainable regeneration, social change
25	24	1	2010	ecosystem service, understanding linkage, export agriculture
26	23	0.948	2008	industrial-flight hypothesis, adversarial legalism, Canadian environmental policy
27	23	0.953	2005	agri-environmental policy, farmer participation, conservation-oriented thinking
28	22	0.99	2016	Chinese cities, transitional china, polluting industries
29	20	1	2013	ecosystem service, ecosystem services framework, ecosystem services assessment
31	13	0.99	2007	policy learning, spatial indeterminacy, environmental knowledge

## Appendix B

**Table A2:** The burst strength of keywords (part). The blue line represents the general time period and the red line represents the specific time period for keywords burst.

Keyword	Year	Strength	Begin	End	2000–2019
climate change adaptation	2000	5.8845	2015	2019	▂▂▂▂▂▂▂▂▂▂▂▂▂▂▂ ▃▃▃▃▃
legitimacy	2000	5.2736	2015	2019	▂▂▂▂▂▂▂▂▂▂▂▂▂▂▂ ▃▃▃▃▃
eco innovation	2000	11.9291	2015	2019	▂▂▂▂▂▂▂▂▂▂▂▂▂▂▂ ▃▃▃▃▃
environmental Kuznets curve	2000	5.1234	2015	2019	▂▂▂▂▂▂▂▂▂▂▂▂▂▂▂ ▃▃▃▃▃
directional distance function	2000	4.2491	2015	2019	▂▂▂▂▂▂▂▂▂▂▂▂▂▂▂ ▃▃▃▃▃
manufacturing industry	2000	6.2783	2016	2019	▂▂▂▂▂▂▂▂▂▂▂▂▂▂▂▂ ▃▃▃▃
africa	2000	5.2156	2016	2019	▂▂▂▂▂▂▂▂▂▂▂▂▂▂▂▂ ▃▃▃▃
epic model	2000	5.7548	2016	2019	▂▂▂▂▂▂▂▂▂▂▂▂▂▂▂▂ ▃▃▃▃
transition	2000	7.2361	2016	2019	▂▂▂▂▂▂▂▂▂▂▂▂▂▂▂▂ ▃▃▃▃
empirical analysis	2000	7.0501	2016	2019	▂▂▂▂▂▂▂▂▂▂▂▂▂▂▂▂ ▃▃▃▃
pollution haven hypothesis	2000	5.1396	2016	2019	▂▂▂▂▂▂▂▂▂▂▂▂▂▂▂▂ ▃▃▃▃
policy implementation	2000	4.708	2016	2019	▂▂▂▂▂▂▂▂▂▂▂▂▂▂▂▂ ▃▃▃▃
calibration	2000	5.4931	2016	2019	▂▂▂▂▂▂▂▂▂▂▂▂▂▂▂▂ ▃▃▃▃
food security	2000	5.1005	2016	2019	▂▂▂▂▂▂▂▂▂▂▂▂▂▂▂▂ ▃▃▃▃
energy consumption	2000	6.555	2016	2019	▂▂▂▂▂▂▂▂▂▂▂▂▂▂▂▂ ▃▃▃▃
urbanization	2000	4.7894	2016	2019	▂▂▂▂▂▂▂▂▂▂▂▂▂▂▂▂ ▃▃▃▃
renewable energy	2000	4.1697	2017	2019	▂▂▂▂▂▂▂▂▂▂▂▂▂▂▂▂▂ ▃▃▃
public opinion	2000	6.1625	2017	2019	▂▂▂▂▂▂▂▂▂▂▂▂▂▂▂▂▂ ▃▃▃
environmental innovation	2000	4.7018	2017	2019	▂▂▂▂▂▂▂▂▂▂▂▂▂▂▂▂▂ ▃▃▃
firm performance	2000	5.8218	2017	2019	▂▂▂▂▂▂▂▂▂▂▂▂▂▂▂▂▂ ▃▃▃
foreign direct investment	2000	4.4644	2017	2019	▂▂▂▂▂▂▂▂▂▂▂▂▂▂▂▂▂ ▃▃▃
carbon tax	2000	5.0782	2017	2019	▂▂▂▂▂▂▂▂▂▂▂▂▂▂▂▂▂ ▃▃▃
undesirable output	2000	9.5609	2017	2019	▂▂▂▂▂▂▂▂▂▂▂▂▂▂▂▂▂ ▃▃▃
stakeholder engagement	2000	5.396	2017	2019	▂▂▂▂▂▂▂▂▂▂▂▂▂▂▂▂▂ ▃▃▃
supply chain	2000	4.7938	2017	2019	▂▂▂▂▂▂▂▂▂▂▂▂▂▂▂▂▂ ▃▃▃
pm25	2000	5.1365	2017	2019	▂▂▂▂▂▂▂▂▂▂▂▂▂▂▂▂▂ ▃▃▃
carbon dioxide emission	2000	8.548	2017	2019	▂▂▂▂▂▂▂▂▂▂▂▂▂▂▂▂▂ ▃▃▃
shadow price	2000	4.7398	2017	2019	▂▂▂▂▂▂▂▂▂▂▂▂▂▂▂▂▂ ▃▃▃
natural gas	2000	6.2341	2017	2019	▂▂▂▂▂▂▂▂▂▂▂▂▂▂▂▂▂ ▃▃▃
resource based view	2000	5.1032	2017	2019	▂▂▂▂▂▂▂▂▂▂▂▂▂▂▂▂▂ ▃▃▃
productivity growth	2000	5.0112	2017	2019	▂▂▂▂▂▂▂▂▂▂▂▂▂▂▂▂▂ ▃▃▃
eco efficiency	2000	6.5072	2017	2019	▂▂▂▂▂▂▂▂▂▂▂▂▂▂▂▂▂ ▃▃▃
spillover	2000	9.2259	2017	2019	▂▂▂▂▂▂▂▂▂▂▂▂▂▂▂▂▂ ▃▃▃
crop yield	2000	6.1625	2017	2019	▂▂▂▂▂▂▂▂▂▂▂▂▂▂▂▂▂ ▃▃▃
empirical evidence	2000	15.3439	2017	2019	▂▂▂▂▂▂▂▂▂▂▂▂▂▂▂▂▂ ▃▃▃
panel data	2000	11.1817	2017	2019	▂▂▂▂▂▂▂▂▂▂▂▂▂▂▂▂▂ ▃▃▃
technical change	2000	5.4542	2017	2019	▂▂▂▂▂▂▂▂▂▂▂▂▂▂▂▂▂ ▃▃▃
